# Prevalence of mental disorders and related risk factors in refugees and asylum seekers in Campania

**DOI:** 10.3389/fpsyt.2024.1478383

**Published:** 2024-11-12

**Authors:** Luigi Giuliani, Paola Bucci, Raffaele Bracalenti, Giulia Maria Giordano, Matteo Conenna, Giulio Corrivetti, Davide Palumbo, Andrea Dell’Acqua, Federica Piras, Giovanna Storti, Verdiana Abitudine, Roberta Di Lieto, Letizia Sandolo, Chiara Schiavitelli, Alice Mulè, Pierpaola D’Arista, Armida Mucci, Silvana Galderisi

**Affiliations:** ^1^ Department of Mental and Physical Health and Preventive Medicine, University of Campania “Luigi Vanvitelli”, Naples, Italy; ^2^ Istituto Psicoanalitico per le Ricerche Sociali (IPRS), Rome, Italy; ^3^ Department of Mental Health of Salerno, Local Health Center of Salerno, Salerno, Italy; ^4^ Neuropsychiatry Laboratory, Department of Clinical Neuroscience and Neurorehabilitation, Istituto di Ricovero e Cura a Carattere Scientifico (IRCCS) Santa Lucia Foundation, Rome, Italy; ^5^ Integrated Area for Fragility, Local Health Center of Salerno, Salerno, Italy

**Keywords:** refugees, asylum seekers, mental health, health policy, prevention, early intervention

## Abstract

**Introduction:**

In recent years, the increasing presence of refugees and asylum seekers displaced from their country of origin, determined significant social, economic, humanitarian and public health implications in host countries, including Italy. These populations are exposed to several potential stressful experiences which make them vulnerable to psychological distress. In fact, the majority of studies addressing the topic found a higher prevalence of mental disorders, especially post-traumatic stress disorder and major depressive disorder, in refugees and asylum seekers with respect to the general population. However, heterogeneous prevalence rates have been reported among studies, due to methodological factor as well as to the impact of a variety of risk factors related to stressful experiences lived in the country of origin, during the migration journey and in the host country.

**Objectives:**

The aim of the present study was to assess the prevalence of the main psychiatric diagnoses in a large group of adult refugees and asylum seekers (N=303) in the reception centers of two provinces of the Campania region, as well as to investigate the impact of potential risk factors on the occurrence of psychiatric disorders.

**Methods:**

The diagnosis of psychiatric disorders and the identification of subjects at high risk to develop psychosis were carried out by means of structured diagnostic interviews. The following variables were explored as potential risk/protective factors to the occurrence of psychiatric disorders: socio-demographic variables, migration status (refugees/asylum seekers) and characteristics of the reception center,assessed by means of an *ad hoc* questionnaire; cognitive indices assessed by using standardized neuropsychological tests; traumatic experiences and level of political terror in the country of origin, assessed by means of reliable and valid self-report questionnaires.

**Results:**

At least one mental disorder was found in 29.7% of the sample. Most prevalent diagnoses were depressive disorders, anxiety disorders and PTSD. Women showed, with respect to men, a higher prevalence of anxiety disorders, higher trauma levels, and came from more at-risk countries. Higher trauma levels, better cognitive abilities and unemployment and refugee status were associated to the presence of a current psychiatric disorder in the whole sample.

**Conclusions:**

Our findings showed a higher prevalence of depressive disorders and PTSD in the sample of refugees and asylum seekers with respect to the general population and highlighted the role of potential risk factors whose identification may guide the implementation of preventive strategies and early treatments in these people.

## Introduction

Migration is a persistent phenomenon since the 19^th^ century (1). At the end of 2022, the United Nations estimated the number of international migrants worldwide at 295 million, corresponding to 3.6% of the world population (2). Out of these, 108.4 million are forced migrants and, in particular, 29.4 million are refugees, 5.4 asylum seekers, and 62.5 are internal displaced persons, while the remaining 11% are Palestinians refugees or Venezuelan displaced abroad. An even broader migration pattern has been reported in Italy, due to its geographical position. As a matter of fact, at the end of 2023, Italy registered the entrance of 157,652 people, with an increase of migration flows as compared to 2022 and 2021, when 105,131 and 67,477 migrants respectively arrived in the country (3).

Refugees and asylum seekers are exposed to several potential stressful and traumatic experiences which can occur before migration, during the migration journey, as well as after the arrival in the host nation, which make them vulnerable to psychological distress and to the occurrence of mental disorders (4). One review and one meta-analysis on the topic found that refugees and asylum seekers, with respect to the general population, have a higher overall lifetime prevalence of post-traumatic stress disorder (PTSD) and major depressive disorder but not of anxiety disorders and psychosis (5, 6). However, discrepant findings have also been reported, such as a higher prevalence reported also for anxiety disorders (7), lack of difference in the prevalence of depression (8) and higher prevalence of psychosis (9). A constant issue emerging from reviews and meta-analyses addressing this topic is the wide variability in the prevalence rate of mental disorders among studies (5–8, 10, 11). The same heterogeneous picture arises from studies carried out in samples of refugees and asylum seekers hosted in Italian regions, in which prevalence rates ranged from 17% to 40% for PTSD (12–16), from 7% to 15% for depressive disorders or depressive symptoms (13, 15–17), from 12% to 48% for anxiety disorders or anxiety symptoms (13, 16), and from 11.8% to 30.8% for psychosis (16, 18).

Factors responsible of such a variability can be grouped in two main categories: a) methodological differences among studies and b) heterogeneity among socio-demographic characteristics and experiences of refugees and asylum seekers, which represent risk factors or mediators in the development of certain mental disorders.

Methodological factors mainly impacting prevalence rates of mental disorders in refugees and asylum seekers include: 1) sample size, as studies including more than 200 subjects show lower rates of mental disorders and lower variability among results (19, 20), although the impact of the population dimension on prevalence rates seems limited to PTSD and depression and does not impact anxiety disorders (6); 2) sampling methods, with studies using nonrandom sampling showing higher rates of PTSD and depression (20); 3) type of assessment instruments, since lower rates of mental disorders are reported in studies using structured diagnostic interviews vs. those using self-report questionnaires or screening tools (6, 11, 19, 21); 4) lack of cross-cultural adaption and/or validation of diagnostic and assessment tools used in some studies, which may generate misunderstanding of the situations presented as exemplification of distress or to describe symptoms (7, 19); 5) lack of cultural mediators assistance in the diagnostic assessment, which is reported associated to a lower frequency of mental disorders (6); 6) lack of distinction in many studies between refugees and asylum seekers, although such differentiation has been recommended by the WHO (22), since the two groups do not receive the same level of protection and benefits in the host country and may have been exposed to different experiences of stressors in their country of origin (21, 23).

In addition to the above-mentioned potential methodological biases, a true heterogeneity in prevalence rates among different populations of refugees and asylum seekers may be related to differences on factors impacting the development of mental disorders, such as some socio-demographic characteristics and/or the experiences lived before, during and after the migration journey. Among-socio-demographic variables, female gender and higher levels of education have been found associated to higher risk to develop psychiatric disorders in these populations in some but not all studies; in particular, higher prevalence rates of PTSD (6) or anxiety (10) have been reported, as well as a lack of gender differences (24), while higher levels of education have been found to affect prevalence rate of depression (8) or psychiatric symptoms (25). Pre-migration factors related to the country of origin, and to the exposure to potential traumatic events, such as torture and violence, violation of human rights, presence of war conflicts and their duration, as well as separation from family or close friends, have been reported among factors increasing the risk to develop mental disorders in refugees and asylum seekers (4, 10, 14, 21, 26–29). Risk factors related to traumatic events occurring during the migration journey include physical harm, violence and extortion or having witnessed violent events perpetrated on others (4). Post-migration experiences may also represent important risk/protective factors for the development of mental disorders. They include: displacement duration, which has been found associated to lower rates of depression or PTSD in some studies (10, 11) but not in others (6); unemployment, financial difficulties, weak social network and weak social integration, discrimination and uncertainty regarding residence status, which all result associated to higher rates of mental disorders (19, 21, 30, 31). Post-migration factors are often different depending on the host country; this may be related to differences among host countries such as immigration policy strategies (32, 33), level of income – which may have an impact on some of the above-reported risk factors such as unemployment and financial difficulties, and public attitudes toward immigration among countries (34). In line with the latter hypothesis, in a sample of refugees and asylum seekers living in Genoa (Nort-West of Italy), community resilience was identified among protective factors against the effects of mental disorders on well-being (17).

Within this framework, the management of the mental health of refugees and asylum seekers is an important issue for the mental health policies of the host countries, which needs to be considered in the development and organization of mental health interventions and treatment services. In addition, it is crucial to focus on the identification and management of potential risk factors in order to reduce the prevalence rate of mental disorders in refugees and asylum seekers by adopting early interventions and preventive strategies representing main goals to be implemented in psychiatry (35–41). Given the broad migration pattern in Italy and the potential impact of specific aspects of the host countries in the development of psychiatric disorders in refugees and asylum seekers, it could be of interest to assess frequency and correlates of psychiatric disorders in forced migrants hosted in an Italian catchment area. Findings reported in the specific context of Italy didn’t provide a clear and reliable picture so far as they were in part affected by the above-reported methodological limitations such as the inclusion of relatively small sample size (12, 14), the definition of diagnoses by means of self-report questionnaires (14, 16), the retrospective study design (15, 16), the assessment of prevalence rates only for some psychiatric disorders (12, 14) or only in subgroups of refugees and asylum seekers such as those with psychological distress as assessed by means of a self-report questionnaire (12). In the light of these research gaps, in the present study we investigated prevalence rates of main psychiatric disorders and their risk/protective factors in a sample of refugees and asylum seekers hosted in two Campania provinces, Salerno and Avellino. We also investigated the prevalence of psychotic disorders, to offer a contribution in clarifying this point – given the discrepant findings provided so far by the few studies exploring it in the Italian context (16, 18) – as well as the prevalence of subjects at high-risk for psychosis, to explore the need to implement prevention strategies for psychotic disorders. Main methodological biases reported in the literature were addressed, by including a large sample size, assessing psychiatric diagnoses by means of a structured diagnostic interview and distinguishing between refugees and asylum seekers. Variables investigated as potential protective/risk factors were chosen among those more frequently reported in association with psychiatric disorders in refugees and asylum seekers in the above-reported literature, and included socio-demographic indices, migration status (refugees/asylum seekers), characteristics of the reception center, trauma levels and political terror in the country of origin. In addition, the impact of cognitive functioning on the occurrence of psychiatric disorders was explored, particularly with regard to cognitive domains whose deficit may have an impact on psychosocial functioning (42, 43). We aimed at addressing the following questions: 1) Are the prevalence rates of psychiatric disorders in refugees and asylum seekers hosted in Italy higher with respect to the general population? If yes, is this true for all psychiatric diagnoses or just for some of them? We hypothesized to find higher prevalence rates at least for some of the explored diagnoses, such as depression and PTSD, as reported in the majority of papers (5, 6), but probably with lower percentages than those reported in studies biased by small sample size or by the use of self-report questionnaires to perform diagnoses. 2) Is the occurrence of psychiatric disorders in refugees and asylum seekers hosted in Italy influenced by risk factors such as socio-demographic characteristics, cognitive functioning or stressful events occurring in different stages of the migration process? We expected to confirm the associations of psychiatric diagnosis with the risk factors most frequently reported in the literature, such as female gender and levels of trauma, the latter especially in refugees vs. asylum seekers, since the formers may have been exposed to more stressful experiences in their country of origin.

## Methods

### Subjects

Study participants were recruited in second levels reception centers localized in two Campania provinces (Salerno and Avellino) hosting, altogether, about 1,200 refugees and asylum seekers. According to the Italian Law No. 173/2020, the reception centers are called “Reception and integration system” (SAI), which replaced the “Protection system for holders of international protection and for unaccompanied foreign minors” (SIPROIMI) which, in turn, replaced the “Protection system for asylum seekers and refugees” (SPRAR). Subjects were also recruited in “Extraordinary Reception Centers” (CAS), which are temporary facilities to be opened in case of “substantial and close arrivals of applicants that cannot be accommodated through the ordinary system” (Legislative Decree 142/2015, art. 11). The aforementioned legislation also applies to the reception centers of the two provinces involved in the present study. Therefore, the asylum situation in the region of investigation provides a good representation of that of the entire country.

Inclusion criteria were: at least 18 years of age; ability to provide an informed consent; and adequate language skills (Italian and/or English) for understanding informed consent and study interviews (or, in alternative, presence of a cultural mediator).

All patients signed a written informed consent to participate in the study after receiving a comprehensive explanation of its procedures and goals. Confidentiality was strictly maintained by anonymizing the data and storing it in encrypted files. Access to the data was limited to authorized research personnel, and participants were assured that their personal information would not be shared or linked to their responses. The study was approved by the Ethics Committee of the Integrated Area for Fragility of the Local Health Center of Salerno (approval number 233817 of the 03/10/2019).

### Study procedures

Recruitment took place from February 1^st^, 2020, to October 11, 2021. The adequately trained staff of the reception centers involved in the study, provided to: a) select adults with adequate language skills (Italian and/or English) for understanding informed consent and study interviews questions and to answer them; b) explain the study protocol to the selected subjects; c) invite them to participate in the study. All patients who agreed to be included in the study signed a written informed consent after receiving a further comprehensive explanation of study procedures and goals by the researchers.

Enrolled subjects completed the assessments in two days following the schedule below: collection of socio-demographic and migration status information, psychopathological evaluation and cognitive assessment on day 1; diagnostic evaluation on day 2.

### Clinical and psychopathological evaluations

An *ad hoc* questionnaire was developed in order to collect socio-demographic data (gender, age, education, employment), information on the migration status (refugee/asylum seeker) and type of reception center (less/more than 40 people hosted; placed in an urban center with less/more than 10.000 inhabitants).

The Mini-International Neuropsychiatric Interview (MINI) (44) was used for the diagnosis of mental disorders according to the criteria of DSM-IV and ICD-10. The MINI has shown good internal consistency, with Cronbach’s alpha values ranging between 0.79 and 0.90 across different diagnostic categories.

The identification of subjects at high-risk for psychosis was performed using the Comprehensive Assessment of At-Risk Mental States (CAARMS) (45), a semi-structured clinical interview designed to measure attenuated psychotic symptoms. It consists of 27 items (i.e. “Have you noticed any changes in your thoughts, such as thinking about things that don’t make sense?”) rated in terms of intensity and frequency/duration clustered in 7 subscales: positive symptoms, cognitive change, emotional disturbance, negative symptoms, behavioral change, motor/physical changes, general psychopathology. A CAARMS severity score (total-CAARMS) can be extracted by summing the product of the global rating scale score (0-6, where 0 is no symptoms) and the frequency score (0-6, where 0 = has never occurred) of the subscales. The CAARMS demonstrates excellent internal reliability, with Cronbach’s alpha reported at 0.93.

The Hopkins Symptom Checklist-25 (HSCL-25) (46) is a self-report questionnaire consisting of 25 items (i.e. “In the past week, how often have you felt suddenly scared for no reason?”) and exploring symptom severity with regard to anxiety and depression rated on a 4-point scale (from 1=not at all, to 4= extremely). The total score was used for the assessment of depressive and anxiety symptoms. The HSCL-25 has shown high internal consistency in various populations, with Cronbach’s alpha values ranging from 0.87 to 0.90.

### Assessment of traumatic experiences

Traumatic experiences were evaluated with the Harvard Trauma Questionnaire post-traumatic stress checklist (HTQ-16) (47), a self-report questionnaire widely used in refugees and asylum seekers, assessing 16 post-traumatic symptoms (i.e. “Have you had recurrent thoughts or memories of the most hurtful or terrifying events?”) rated on a 4-point scale (from 1=not at all, to 4=extremely). Symptom severity was computed by averaging the 16 scores. The HTQ-16 demonstrates strong internal reliability, with Cronbach’s alpha ranging from 0.89 to 0.92 for the PTSD symptom checklist.

The state inflicted political terror in countries of origin was measured by the Political Terror Scale (PTS) (48), which is an annual measure of political terror in a nation based on a 5-level “terror scale”. A score > 4 indicates the presence of political terror in the country of origin.

### Cognitive assessment

To assess global cognition, and to minimize the influence of linguistic, cultural and educational differences, we used 3 subtests of the Wechsler Adult Intelligence Scale fourth edition (WAIS-IV) (49), “block design”, “puzzles”, “matrix reasoning”. Combining together the scores of these three subtests, we obtained the perceptual reasoning index score, representing a measure of one of the main components of human intelligence. The WAIS-IV has demonstrated high internal consistency across its subtests, with Cronbach’s alpha values ranging between 0.88 and 0.97.

The Trail Making Test-A (TMT-A) (50), the Digit Symbol Coding (50) and the Verbal fluency (50) were used to assess speed of processing; the difference between Trail Making Test-B (TMT-B) (50) minus TMT-A score was used as an index of executive functions. The Trail Making Test, digit symbol coding and verbal fluency tasks showed moderate to good internal consistency, with Cronbach’s alpha values reported between 0.70 and 0.90, depending on the population.

Social cognition was assessed by means of the Facial Emotion Identification Test (FEIT) which has shown moderate to good internal consistency, with Cronbach’s alpha reported between 0.75 and 0.85 (51).

### Training of the researchers

The training of all the researchers was conducted by expert psychologists and psychiatrists working at the Department of Mental and Physical Health and Preventive Medicine of the University of Campania “Luigi Vanvitelli”. Three psychologists were trained for the neurocognitive assessment, while eight trainees in psychiatry (attending the third or the fourth year of course) were trained for the structured diagnostic interviews (MINI and CAARMS).

The inter-rater reliability was formally evaluated by Cohen’s kappa for the MINI and the CAARMS. An excellent inter-rater agreement was found for both interviews: Cohen’s kappa was 0.92 for the former and 0.81 for the latter.

### Statistical analysis

The minimum sample size was calculated setting the critical value associated with the confidence level (Z for 95%) at 1.96, the desired margin of error at 0.05 and the expected prevalence at 0.10–0.15, according to the WHO report on the global prevalence of mental disorders in general population.

The prevalence of mental disorders in the study sample as well as the frequency of each mental disorder were calculated.

All cognitive indices were transformed into z-scores. Composite scores were calculated for global cognition (mean of the 3 WAIS-IV subtests z-scores), for the cognitive domain “Speed of processing” (mean of TMT-A, Symbol Coding and Category fluency z-scores) and for executive functions (TMT-B minus TMT-A z-scores). Values of TMT-A and TMT-B were inverted, because a lower score at these tests correspond to a higher cognitive performance, differently from the other cognitive indices.

Analyses of variance (ANOVA) were performed to investigate group differences between subjects with vs. without at least one psychiatric disorder, between refugees vs. asylum seekers and between men vs. women for the following variables: speed of processing, executive functions, social and global cognition scores, traumatic events, anxious and depressive symptoms. Group differences in the frequency of psychiatric disorders were explored by means of χ² square test.

Pearson’s correlation analyses were conducted to investigate the relationship between HSCL-25 psychopathological index and the other study variables.

A logistic regression analysis was performed to identify factors associated to the presence of current mental disorder. The presence or the absence of a psychiatric diagnosis was selected as dependent variable. Gender, education, occupation status (employed/unemployed), migration status (refugees/asylum seekers), number of people hosted in the reception center (high/low), type of urban center (with high/low number of inhabitants), trauma levels (average score at the HTQ-16), social cognition (FEIT total number of correct answers), cognitive indices and political terror in the country of origin (score > 4 at the Political Terror Scale) were selected as independent variables. Multicollinearity among the independent variables was assessed using the Variance Inflation Factor (VIF). Variables with a VIF greater than 5 were considered to indicate potential multicollinearity. An *a priori* power analysis was conducted using Cohen’s f² to assess the adequacy of the sample size for detecting medium effect sizes in logistic regression models.

Statistical significance was fixed at p<.05 and SPSS v. 25 software was used for all the above-mentioned analyses.

## Results

### Characteristics of study sample

We estimated that the minimum sample size should be 139-196 subjects, setting the prevalence of mental disorders in general population respectively at 10 and 15%.

One thousand two hundred people hosted in the CAS or SPRAR/SIPROIMI structures sited in the and Avellino and Salerno provinces were screened. Three hundred and three subjects (241 males, 62 females) met inclusion criteria and were enrolled in the study. Their demographic and clinical characteristics are reported in **Table 1**. Mean age of the participants was 28 ± 6.58 years, mean education was 9.4 ± 4.11; 103 of them (34%) were employed at the time of the screening (72% of which did menial jobs, mainly farm hands); 246 subjects (81%) were asylum seekers, while 57 (19%) were refugees; 197 subjects (65%) came from countries with a high-risk for traumatic events (conflicts, war, political terror, etc.) as indicated by a score >4 at the Political Terror Scale.

**Table 1:** Characteristics of study sample (N=303).

	N	%
Males	241	79.5
Females	62	20.5
	Mean ± SD	Yes (%)
Age (years)	28 **±** 6.58	
Education	9.4 **±** 4.11	
Employment		103 (33.99)
Refugee status		57 (19)
Political Terror Scale > 4		197 (65)
CAARMS positive for high-risk status		6 (2)
MINI positive for psychiatric disorder		90 (29.7)
Harvard Trauma Questionnaire mean score	1.45 **±** 0.50	
Hopkins Symptoms Checklist total score	36 **±** 12.44	
FEIT total correct answers	29 **±** 9.84	
TMT-A	70 **±** 49.9	
TMT-B	158 **±** 76.8	
Symbol Coding - correct answers	30.13 **±** 13.06	
Verbal Fluency- number of categories	11.26 **±** 4.04	
WAIS Cubes	20.45 **±** 10.32	
WAIS Matrices	7.27 **±** 4.25	
WAIS Puzzles	6.49 **±** 3.24	

CAARMS, Comprehensive Assessment of at-risk Mental States; MINI, MINI-International Neuropsychiatric Interview; FEIT, Facial Emotion Identification Test; TMT, Trial Making Test; WAIS, Wechsler Adult Intelligence Scale.

Data on the nationality of study participants are provided in **Table 2**; the most frequent country of origin was Nigeria (41%), followed by Pakistan (28%), Bangladesh and Gambia (9%).

**Table 2:** Country of origin of study participants.

	Attendance	Percentage	Cumulative percentage
Afghanistan	7	2,3	2,3
Albania	2	,7	3,0
Bangladesh	26	8,6	11,6
Burkina Faso	1	,3	11,9
Camerun	3	1,0	12,9
Ivory Coast	13	4,3	17,2
Egypt	3	1,0	18,2
Gambia	26	8,6	26,7
Ghana	10	3,3	30,0
Guinea	11	3,6	33,7
Lebanon	3	1,0	34,7
Liberia	1	,3	35,0
Mali	14	4,6	39,6
Morocco	4	1,3	40,9
Nigeria	125	41,3	82,2
Pakistan	28	9,2	91,4
Senegal	12	4,0	95,4
Sierra Leone	2	,7	96,0
Syria	3	1,0	97,0
Somalia	2	,7	97,7
Sudan	3	1,0	98,7
Togo	3	1,0	99,7
Tunisia	1	,3	100,0
**Total**	303	100,0	

### Frequency of mental disorders in the study sample

Ninety subjects (29.7%) met criteria for a full-blown diagnosis of a mental disorder, as assessed by the MINI, 75 of them (25% of the sample) had a current psychiatric disorder, while the remaining 15 (17%) had only a psychiatric diagnosis in the medical history with no current disorder. Six subjects (2%) resulted at high-risk for developing psychosis.

The most frequent psychiatric diagnoses were major depressive episode (13.2%; 10.2% of which in the medical history and 3% current), followed by panic disorder (6.3%; 5.6% of which lifetime and 0.7% current), PTSD (6%) and generalized anxiety disorder (6%). The prevalence of any depressive disorder was 16.5% and that of any anxiety disorder was 16.2%. Further details on the frequency of psychiatric disorders in the study sample are reported in **Table 3**.

**Table 3:** Frequency of psychiatric disorders.

Disorder	N	%
Major depressive episode (current)	9	3
Major depr. episode (medical history)	31	10.2
EDM Melancholic Manifestations	1	0.3
Dysthymia	9	3
Suicidal ideation	10	3.3
Current (hypo)manic episode	2	0.7
(Hypo)manic episode (medical history)	4	1.3
Current panic disorder	2	0.7
Lifetime panic disorder	17	5.6
Agoraphobia	6	2
Social phobia	4	1.3
Specific phobia	2	0.7
Ossessive/Compulsive Disorder	0	0
Post-Traumatic stress Disorder	18	6
Alcohol Addiction/Abuse	5	1.7
Substance Addiction/Abuse	3	1
Lifetime Psychotic Disorder	3	1
Current Psychotic Disorder	6	2
Anorexia	0	0
Bulimia	0	0
Generalized Anxiety	18	6
Antisocial	0	0
Somatization Disorder	0	0
hypochondria	0	0
Body Dysmorphism	0	0
Algic Disorder	3	1
Conduct Disorder	0	0
Attention/Hyperactivity	0	0
Adjustment disorder	6	2
Premenstrual Dysphoric	1	0.3
Anxious/Depressive Mixed	5	1.7
**Ultra high risk**	6	2

### Group comparison between subjects with and without psychiatric disorders

Subjects with at least one psychiatric disorder with respect to those with no psychiatric disorders showed higher trauma levels (F=70.59; p<.0001), a better performance on global cognition (F=6.62; p=.011) and social cognition (F=8.22; p=.004), and greater severity of anxiety and depressive symptoms (F=61.84; p<.0001). No significant group difference was found for speed of processing (F=2.41; p=.122), executive functions (F=1.53; p=.192) and political terror scale (F=1.82; p=.178).

### Group comparison between refugees and asylum seekers

No difference on the frequency of mental disorders was observed between refugees and asylum seekers (χ²=1.71; p=.19).

Asylum seekers with respect to refugees showed higher levels of trauma (F= 10.65; p=.001), more severe anxiety and depressive symptomatology (F=7.89, p=.005), higher speed of processing (F=5.61, p=.02) and better global cognition (F=4.12; p=.04).

### Group comparison between male and female subjects

No gender difference was observed on the frequency of mental disorders (χ²=2.04; p=.153). When considering separately the diagnosis more frequently observed in the whole sample, anxiety disorders resulted significantly more frequent in women than in men (17.7% vs. 8.3%, χ²=4.79; p=.03), while no difference was observed in the prevalence rate of depressive disorders and PTSD (19% vs. 16%, χ²=0.46; p=.50 and 6.4% vs. 5.8%, χ²=0.04; p=.85, respectively). As for rate of employment, a significant difference was observed with less employed women compared to men (χ²=13.18; p<.0001). Moreover, women showed, with respect to men, higher levels of political terror in the country of origin (F=21.04; p<.0001), more severe anxiety and depressive symptoms (F=10.05; p=.002), higher trauma levels (F=4.92; p=.03) and better performance on the test exploring social cognition (F=5.79; p=.02).

### Relationship of psychopathology and psychiatric diagnosis with sociodemographic variables, cognitive performance and risk factors for psychiatric disorders

Pearson’s correlation analyses showed that the severity of anxious and depressive symptoms assessed by the HSCL-25 was significantly associated only with the severity of trauma levels, measured with the HTQ-16. No significant associations were found with cognitive indices nor with the political terror scale.

A logistic regression analysis was performed to investigate factors associated with the presence of current mental disorders. Therefore, the 15 subjects who had a psychiatric disorder only in the medical history without any current psychiatric diagnosis were excluded from this analysis. The power analysis revealed that, given the sample size of 288 participants and a significance level of 0.05, the analysis indicated that the power to detect medium-sized effects (f² = 0.15) was sufficient (99.9%). The presence of a current psychiatric diagnosis resulted associated with higher levels of trauma (p<.001), unemployment (p<.008), refugee status (p=.04) and better global cognition (p<.03) (**Table 4**). Multicollinearity diagnostics using the Variance Inflation Factor (VIF) revealed that only two variables (global cognition and speed of processing) exceeded the critical threshold of 5, suggesting potential multicollinearity. This indicates that the relationship between these two variables could have inflated the association of global cognition with the presence of current mental disorders. Indeed, after having removed the processing speed from the predictors, the association between global cognition and the presence of a current psychiatric diagnosis became not significant.

**Table 4:** Factors associated with the presence of a current psychiatric disorder (N=288).

	B	S.E.	P	95% Confidence Interval	
				Inferior	Superior
Intercept	-5.945	1.707	<.001*		
Gender (M vs F)	0.388	0.485	0.423	-0.575	1.336
Education	0.012	0.122	0.921	-0.231	0.251
Unemployment vs employment	-1.163	0.436	0.008*	-2.063	-0.342
Refugee vs asylum seeker	0.914	0.447	0.041*	0.033	1.797
Number of guests (high vs low)	0.124	0.400	0.757	-0.648	0.930
Density of population (high vs low)	-0.009	0.400	0.983	-0.792	0.784
Political Terror	-0.418	0.398	0.294	-1.203	0.364
Trauma levels	2.944	0.441	<.001*	2.133	3.868
Global cognition	1.257	0.576	0.029*	0.143	2.412
Speed of processing	-0.829	0.493	0.093	-1.808	0.137
Executive functions	-0.069	0.212	0.743	-0.502	0.335
Social cognition	0.014	0.022	0.519	-0.029	0.059

*p<0.05.

## Discussion

According to our findings, 29.7% of the people hosted in refugee centers in the Campania provinces of Avellino and Salerno suffered from at least one lifetime full-blown mental disorder and 25% of them had a current mental disorder. In line with other studies (5, 6, 19, 21, 52), we found that the most frequent psychiatric diagnoses were any depressive disorder, any anxiety disorder and PTSD. A higher prevalence with respect to the general population (53–58) was observed in our sample only for depressive disorders (16.5% vs. 12%) and PTSD (6% vs. 3.9%), while a prevalence comparable to that of the general population was observed for the other psychiatric disorders.

The prevalence of PTSD and depressive disorders observed in our sample, although higher with respect to that of the general population, was lower with respect to that reported in other studies (6, 12, 15, 21, 24). This is probably related to methodological factors. In fact, higher prevalence of PTSD and depressive disorders were reported in reviews and meta-analysis including data from studies conducted also in middle- and low-income countries, and/or with heterogeneous methodological quality (6, 21), as well as in studies with sample sizes < 200 (12) or in which diagnosis was not performed by means of diagnostic tools (15). As a matter of fact, prevalence rates similar to those found in our sample were reported in studies including only research conducted in high-income countries and of high methodological quality (i.e., sample size > 200 and assessment by means of diagnostic instruments) (5, 10, 59, 60).

We found a prevalence of psychotic disorders comparable to that of the general population. The literature in this regard is quite heterogeneous as some studies report a comparable or even reduced prevalence in refugees and asylum seekers vs. the general population (5) while others found an increased prevalence (16, 61–64). However, it must be noticed that studies reporting a greater prevalence of psychosis found that this was limited to some ethnic groups or to refugees and asylum seekers in some host countries, or to the migration status of refugees, thus suggesting that the development of a psychotic disorder is related to several factors, including the characteristics of the host countries, which may affect the quantity and quality of social interaction of refugees and asylum seekers. The low number of subjects with lifetime or current psychotic disorder in our sample (N=9) does not allow to further clarify this point.

Six individuals (2%) in our sample resulted at high risk for developing psychosis, a prevalence comparable to that reported in the general population (65–69). So far, this condition was explored in refugees and asylum seekers in a very few numbers of studies reporting no higher prevalence with respect to the general population (70).

In our sample, women showed more severe anxiety and depressive symptoms and a higher prevalence of anxiety disorders with respect to men, while the prevalence of depressive disorder, PTSD and any psychiatric disorder did not show any gender differences. Literature findings in this respect are very heterogeneous, as no gender differences in prevalence have been reported, as well as an increased prevalence in women of anxiety disorders, or depressive disorders, or PTSD (10, 24). These discrepancies may be related to the fact that female gender may not represent per se a risk factor to develop psychiatric disorders in refugees and asylum seekers but be a further element of vulnerability in people exposed to other risk factors, such as traumatic events. As a matter of fact, women in our sample were from more at-risk countries and had higher trauma levels. These factors, in addition to the finding of a better performance on social cognition, which may cause higher levels of frustration related to the difficulty of resettlement, could underlie the greater severity of anxiety and depressive symptoms and the higher frequency of anxiety disorders found in women.

Our findings highlighted the impact of traumatic events on mental health in refugees and asylum seekers. First, we found significantly higher trauma levels in subjects with at least one psychiatric disorder vs. those with no psychiatric disorder. Second, we observed a strong association between levels of trauma and severity of depressive and anxious symptoms as assessed by the HSCL-5. Third, trauma level was the most statistically significant independent variable, among those explored in the logistic regression, resulting associated with the presence of a current psychiatric disorder. These findings are widely supported by the literature, which has shown that trauma plays a central role in the development of psychological distress in the forced migrant populations (4, 12, 52). Refugees and asylum seekers may undergo traumatic experiences both in their country of origin, in the context of the migration process, and in the host country. The role of political terror level in the country of origin has been explored to understand the extent to which it affects the development of psychiatric disorders. Unlike Steel et al. (20), who found a strong association between high levels of PTS and PTSD diagnosis, and a weaker association with depressive disorders, in our sample there was no such positive correlation of PTS with anxiety and depressive symptomatology assessed by means of the HSCL-25 and no significant difference in the PTS scale between subjects with psychiatric disorder and those without. This finding supports the view that traumatic events experienced in the country of origin are not the only stressful factors related to the occurrence of psychiatric disorders, as many other stressful and traumatic events occurring during the migration journey, as well as after the arrival in the host country, can make refugees and asylum seekers vulnerable to psychological distress and to the occurrence of mental disorders. This finding highlights the importance to implement preventive strategies in host countries to manage potential risk factors.

To the best of our knowledge, no study explored so far, the impact of cognitive functioning in the occurrence of psychiatric disorders in a population of recently settled asylum seekers and refugees. Since cognitive functioning and psychopathology are independent but related dimensions, which interact in a transdiagnostic manner (71), we investigated the issue and found that the group of subjects with at least one psychiatric disorder had a better performance in global and social cognition with respect to subjects with no psychiatric disorders. In addition, a better performance on global cognition resulted associated in the linear regression analysis to the presence of a current psychiatric disorder. These findings may sound unexpected, given the well-known presence of cognitive deficit in some psychiatric disorders, mainly schizophrenia and bipolar disorders (72–75). However, it must be noticed that these disorders were poorly represented in our sample, while the most frequent psychiatric diagnoses were depressive disorders, anxiety disorders and PTSD. Additionally, studies exploring whether the association between cognitive ability and mental health (depression, anxiety, and psychological wellbeing) could be accounted for by different categories of risk factors such as socioeconomic status, coping/appraisal and social relationships demonstrated that such association can be partly explained by cognitive-behavioral mechanisms also highlighting the protective influence of socioeconomic status (76). The association of a better cognitive performance in subjects with these psychiatric diagnoses in our sample may be explained by the higher degree of frustration and disappointment of expectations experienced by subjects with better cognitive abilities. This would lead them to be more affected by the stressful events associated with the migratory journey and settlement, thus being more exposed to the development of anxiety and depressive syndromes. This hypothesis is in line with the finding reported in some studies of a higher risk to develop depressive and anxious symptoms in refugees with higher degrees of education, as they might suffer from the downgrading of social status in the host country compared to their qualifications (25, 77), however it may sound quite speculative. As a matter of fact, it must be noticed that the association between cognitive performance and the presence of current mental disorders seems to be influenced by multicollinearity among cognitive variables, in particular global cognition and speed of processing, and that the observed association between global cognition and the presence of a current psychiatric diagnosis became not statistically significant after removing the processing speed from the predictors. Further research exploring the impact of cognitive functioning on the occurrence of psychiatric disorders in these populations is needed to clarify this uncertain point.

In addition to trauma levels and cognitive abilities, the other variables resulting associated in the linear regression to the presence of a current psychiatric disorder, were unemployment and refugee status. Unemployment in itself has been identified as a risk factor for mental disorders and can be an obstacle to a full integration into the new social context (4, 52, 78). As to the refugee status, it may represent a further risk factor probably in relation to the higher risks present in their country of origin, as reported in other studies (6). On the other hand, in line with the findings of some reviews (21, 79) we found that asylum seekers with respect to refugees showed more severe anxiety and depressive symptomatology, which may be related to post-migration factors, such as the uncertainty of their condition, as by definition asylum seeker is a person who hasn’t yet been legally recognized as a refugee and is waiting to receive a decision on their asylum claim, and is not allowed to work in the country until is granted refugee status.

The main strengths of our study are: a) sample size > 200 subjects; b) the assessment of psychiatric diagnosis carried out by means of structured diagnostic interviews; c) the novelty of assessing the risk for developing psychosis and the impact of cognitive performance. The following study limitation have to be acknowledged: a) lack of information on specific traumatic events and displacement duration, which may represent a further risk factor to develop psychiatric disorders; b)lack of information about the length of stay in Italy of study participants; c) although in the study design the presence of cultural mediators was planned when needed, it was not possible to recruit them, especially due to the pandemic, therefore only Italian and English speaking subjects could be included in the study with a possible bias to a lower frequency of mental disorders. In addition, it cannot be excluded that the absence of cultural mediators have impacted the validity of collected data among subjects with limited linguistic skills in Italian or English, although it must be noticed that inclusion criteria required adequate language skills (Italian and/or English) for understanding informed consent and study interviews; d) the recruitment of participants only from specific reception centers in two provinces of the Campania region limits generalizability of our findings.

Overall, our findings suggest the identification of risk factors for the development of psychiatric disorders in a large ample of refugees and asylum seekers, namely higher levels of trauma – related not only to pre-migration stressful events, but also to stressful experiences lived in the host country – better global cognition and unemployment. The identification of such risk factors can be of help in the implementation of preventive strategies and early treatments in forced migrant populations. In particular, our findings suggest the following recommendations: for health policymakers, to invest in interventions targeting the increase of screening for psychiatric disorders during the asylum procedure and the reduction of barriers to mental health care in refugees and asylum seekers living in the community; for mental health professionals, recommendations include a careful assessment of stressful events to which each individual is exposed during all the phases of the migration process in order to work with the patient in focusing on the relationships between current problems and his/her traumatic events and vulnerabilities.

## Data Availability

The raw data supporting the conclusions of this article will be made available by the authors, without undue reservation.
